# Supplementary figures and images for: Comparative transcriptomics characterized the distinct biosynthetic abilities of terpenoid and paeoniflorin biosynthesis in herbaceous peony strains
Source: PeerJ. 2020 Apr 20;8:e8895. doi: 10.7717/peerj.8895 (PMC7179566; doi:10.7717/peerj.8895)

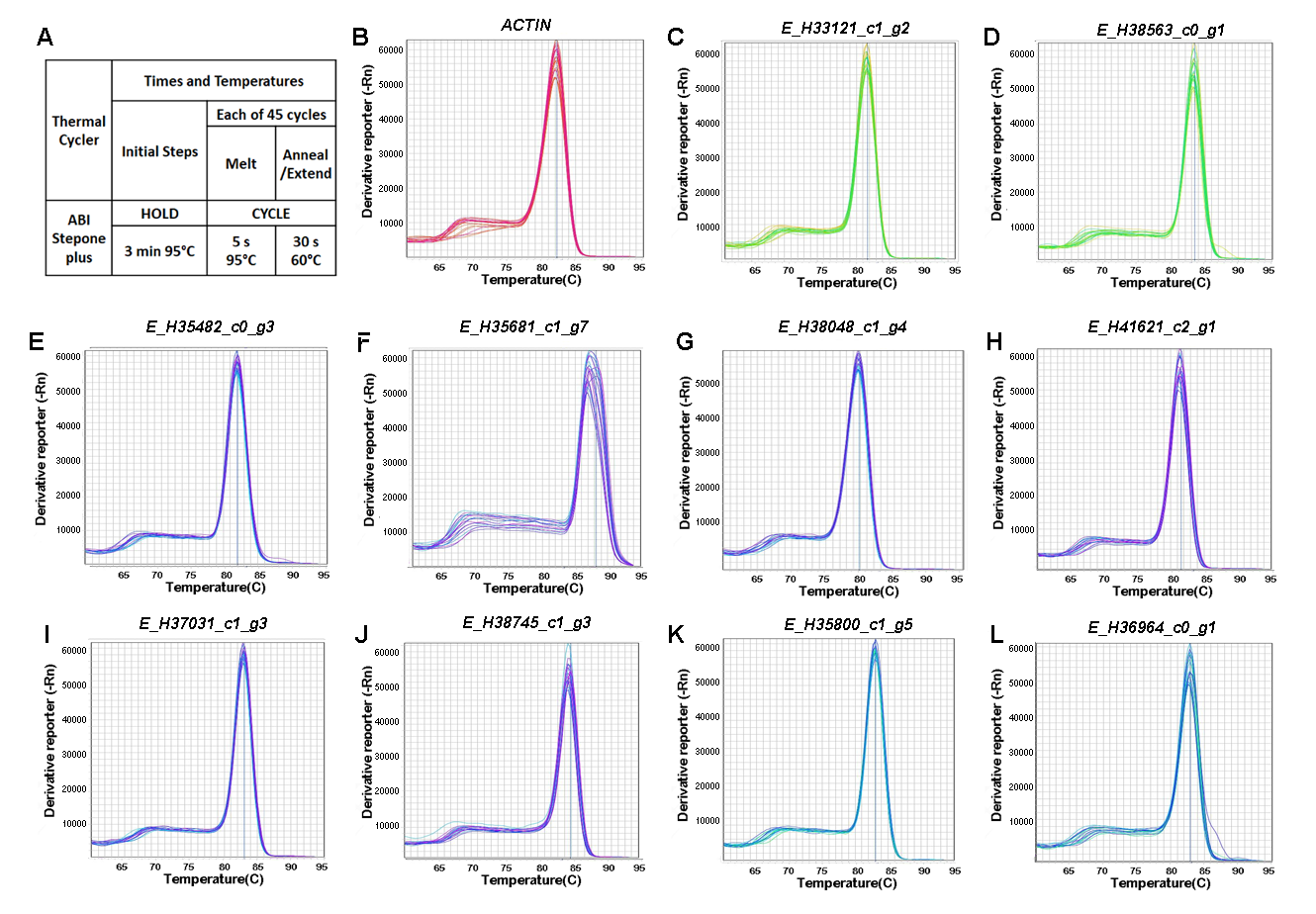

Supplement: Supplemental Information 1 — The melting curves of qRT-PCR primers are given. [file peerj-08-8895-s001.png]

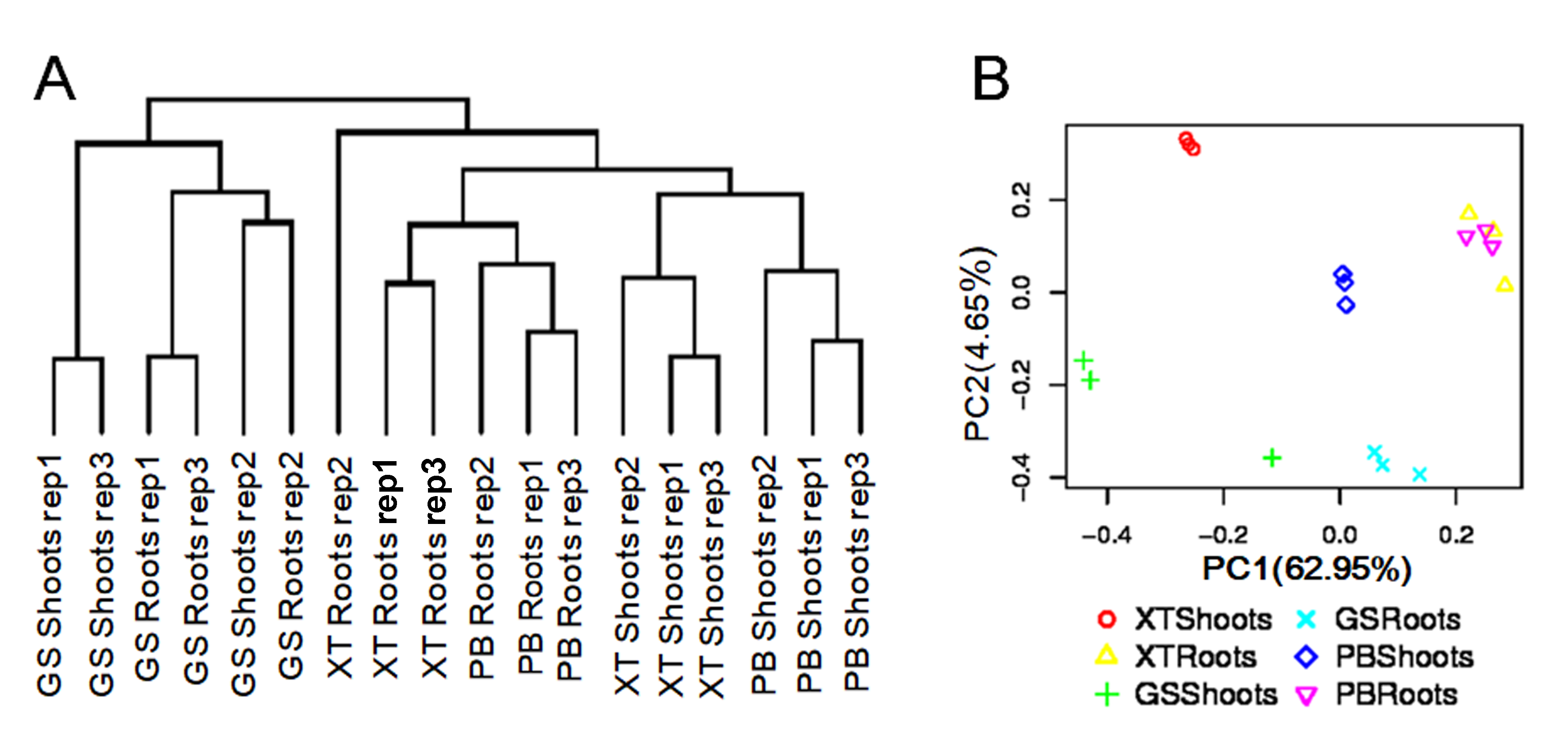

Supplement: Supplemental Information 2 — (A) The hierarchical structure of the gene expression levels. Genes with the FPKM values more than 1 were used for the analysis. (B) Principal component analysis for the genes. [file peerj-08-8895-s002.png]

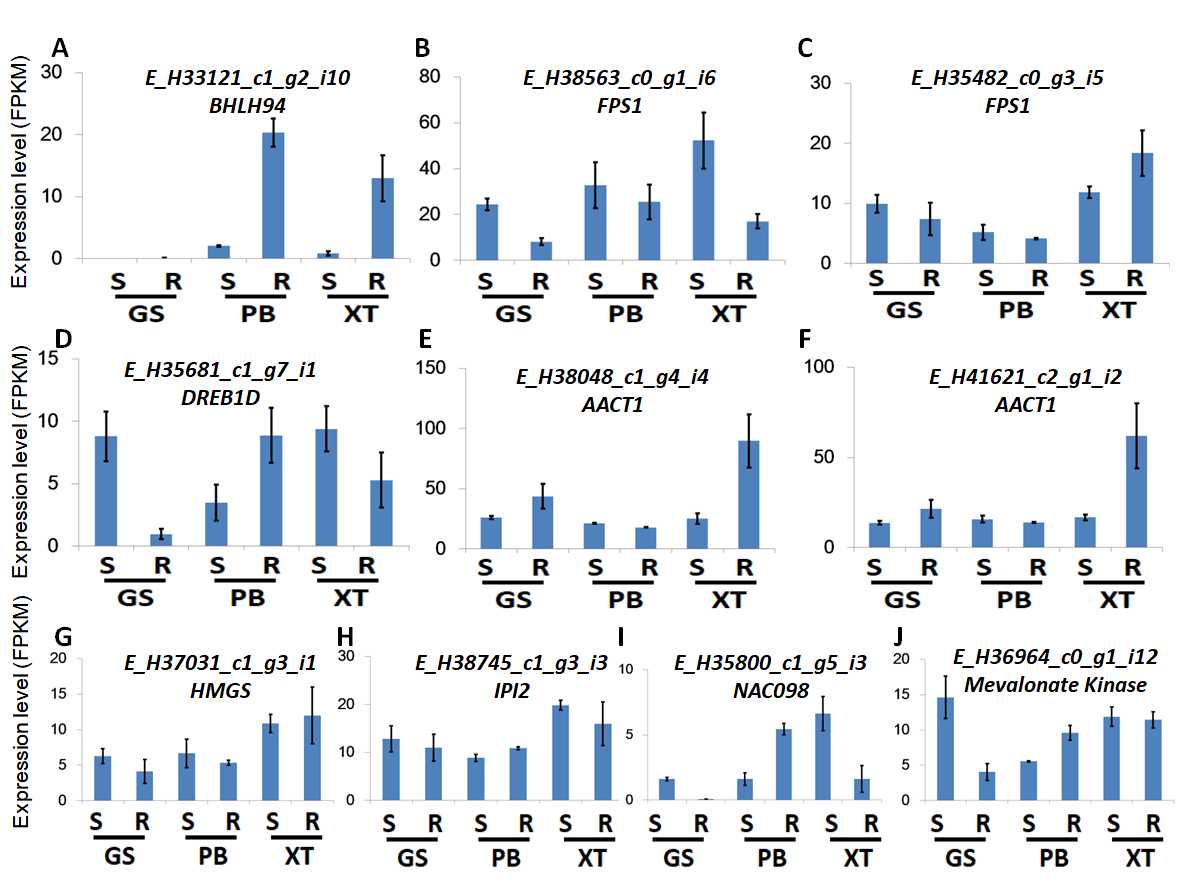

Supplement: Supplemental Information 3 — The normalized FPKM levels of samples were used for presenting expression levels. Bars give standard errors (n = 3). [file peerj-08-8895-s003.png]
